# 4,4′-Azinodibenzoic acid

**DOI:** 10.1107/S1600536809033224

**Published:** 2009-09-05

**Authors:** Qun-Di Yu, Yun-Yu Liu

**Affiliations:** a Food Science and Pharmacy College, Zhejiang Ocean University, Zhoushan 316000, People’s Republic of China; b Department of Chemistry, Northeast Normal University, Changchun 130024, People’s Republic of China

## Abstract

The title compound, C_14_H_10_N_2_O_4_, shows crystallographic inversion symmetry and has one half-mol­ecule in the asymmetric unit. In the crystal, mol­ecules are linked into chains running along the cell diagonal by O—H⋯O hydrogen-bonding inter­actions.

## Related literature

For the use of azodibenzoate-based systems as bridging aromatic carboxyl­ate ligands in coordination networks, see: Chen *et al.* (2008 ▶).
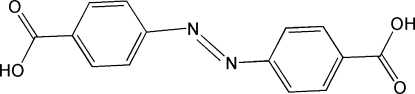


## Experimental

### 

#### Crystal data

C_14_H_10_N_2_O_4_
*M* *_r_* = 270.16
Triclinic, 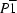

*a* = 3.772 (2) Å
*b* = 6.322 (5) Å
*c* = 12.692 (3) Å
α = 79.323 (5)°
β = 88.199 (4)°
γ = 88.435 (5)°
*V* = 297.2 (3) Å^3^
*Z* = 1
Mo *K*α radiation
μ = 0.11 mm^−1^
*T* = 293 K
0.16 × 0.14 × 0.12 mm

#### Data collection

Bruker SMART APEX CCD area-detector diffractometer
Absorption correction: multi-scan (*SADABS*; Sheldrick 1996 ▶) *T*_min_ = 0.962, *T*_max_ = 0.971
2173 measured reflections
1351 independent reflections
786 reflections with *I* > 2σ(*I*)
*R*_int_ = 0.017

#### Refinement

*R*[*F* ^2^ > 2σ(*F* ^2^)] = 0.040
*wR*(*F* ^2^) = 0.100
*S* = 0.86
1351 reflections
91 parameters
H-atom parameters constrained
Δρ_max_ = 0.19 e Å^−3^
Δρ_min_ = −0.19 e Å^−3^

### 

Data collection: *SMART* (Bruker, 1998 ▶); cell refinement: *SAINT* (Bruker, 1998 ▶); data reduction: *SAINT*; program(s) used to solve structure: *SHELXS97* (Sheldrick, 2008 ▶); program(s) used to refine structure: *SHELXL97* (Sheldrick, 2008 ▶); molecular graphics: *SHELXTL* (Sheldrick, 2008 ▶); software used to prepare material for publication: *SHELXTL*.

## Supplementary Material

Crystal structure: contains datablocks global, I. DOI: 10.1107/S1600536809033224/bt5033sup1.cif

Structure factors: contains datablocks I. DOI: 10.1107/S1600536809033224/bt5033Isup2.hkl

Additional supplementary materials:  crystallographic information; 3D view; checkCIF report

## Figures and Tables

**Table 1:** Hydrogen-bond geometry (Å, °)

*D*—H⋯*A*	*D*—H	H⋯*A*	*D*⋯*A*	*D*—H⋯*A*
O1—H1*A*⋯O2^i^	0.82	1.81	2.6181 (17)	170

Symmetry code: (i) 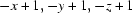.

**Fig. 1.**
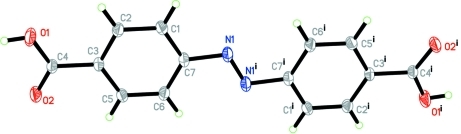
The structure of the title compound, showing the atomic numbering scheme. Displacement ellipsoids are drawn at the 30% probability level. Symmetry code: (i) -x, -y, -z.

## supplementary crystallographic information

### Comment

Azodibenzoate-based systems represent one type of bridging aromatic carboxylate ligand employed in the generation of coordination networks (Chen *et al.*, 2008). There is half a molecule in the asymmetric unit of the title compound (Fig. 1). In the crystal, molecules are linked into chains by O—H···O hydrogen-bonding interactions (Table 2).

### Experimental

A mixture of ZnCl_2_^.^2H_2_O (0.5 mmol), 4,4'-azodibenzoatic acid (0.5 mmol), and H_2_O (500 mmol) was heated at 140 *^o^*C for 3 days. After the mixture was slowly cooled to room temperature, pale yellow crystals of the title compound were yielded (22% yield).

### Refinement

All H atoms on C atoms were positioned geometrically (C—H = 0.93 Å) and refined as riding, with *U*_iso_(H)=1.2*U*_eq_(carrier).

### Crystal data

**Table:**

C_14_H_10_N_2_O_4_	*Z* = 1
*M**_r_* = 270.16	*F*(000) = 140
Triclinic, *P*1	*D*_x_ = 1.509 Mg m^−^^3^
Hall symbol: -P 1	Mo *K*α radiation, λ = 0.71073 Å
*a* = 3.772 (2) Å	Cell parameters from 1351 reflections
*b* = 6.322 (5) Å	θ = 3.0–29.0°
*c* = 12.692 (3) Å	µ = 0.11 mm^−^^1^
α = 79.323 (5)°	*T* = 293 K
β = 88.199 (4)°	Block, pale yellow
γ = 88.435 (5)°	0.16 × 0.14 × 0.12 mm
*V* = 297.2 (3) Å^3^	

### Data collection

**Table:**

Bruker SMART APEX CCD area-detector diffractometer	1351 independent reflections
Radiation source: fine-focus sealed tube	786 reflections with *I* > 2σ(*I*)
graphite	*R*_int_ = 0.017
φ and ω scans	θ_max_ = 29.0°, θ_min_ = 3.3°
Absorption correction: multi-scan (*SADABS*; Sheldrick 1996)	*h* = −5→4
*T*_min_ = 0.962, *T*_max_ = 0.971	*k* = −8→5
2173 measured reflections	*l* = −17→17

### Refinement

**Table:**

Refinement on *F*^2^	Primary atom site location: structure-invariant direct methods
Least-squares matrix: full	Secondary atom site location: difference Fourier map
*R*[*F*^2^ > 2σ(*F*^2^)] = 0.040	Hydrogen site location: inferred from neighbouring sites
*wR*(*F*^2^) = 0.100	H-atom parameters constrained
*S* = 0.86	*w* = 1/[σ^2^(*F*_o_^2^) + (0.0555*P*)^2^] where *P* = (*F*_o_^2^ + 2*F*_c_^2^)/3
1351 reflections	(Δ/σ)_max_ < 0.001
91 parameters	Δρ_max_ = 0.19 e Å^−^^3^
0 restraints	Δρ_min_ = −0.19 e Å^−^^3^

### Special details

**Table:**

Geometry. All e.s.d.'s (except the e.s.d. in the dihedral angle between two l.s. planes) are estimated using the full covariance matrix. The cell e.s.d.'s are taken into account individually in the estimation of e.s.d.'s in distances, angles and torsion angles; correlations between e.s.d.'s in cell parameters are only used when they are defined by crystal symmetry. An approximate (isotropic) treatment of cell e.s.d.'s is used for estimating e.s.d.'s involving l.s. planes.
Refinement. Refinement of *F*^2^ against ALL reflections. The weighted *R*-factor *wR* and goodness of fit *S* are based on *F*^2^, conventional *R*-factors *R* are based on *F*, with *F* set to zero for negative *F*^2^. The threshold expression of *F*^2^ > σ(*F*^2^) is used only for calculating *R*-factors(gt) *etc*. and is not relevant to the choice of reflections for refinement. *R*-factors based on *F*^2^ are statistically about twice as large as those based on *F*, and *R*- factors based on ALL data will be even larger.

### Fractional atomic coordinates and isotropic or equivalent isotropic displacement parameters (Å^2^)

**Table:**

	*x*	*y*	*z*	*U*_iso_*/*U*_eq_	
C1	0.0410 (4)	−0.0391 (3)	0.23081 (12)	0.0356 (4)	
H1	−0.0514	−0.1767	0.2450	0.043*	
C2	0.1284 (4)	0.0608 (3)	0.31492 (12)	0.0343 (4)	
H2	0.0924	−0.0090	0.3855	0.041*	
C3	0.2692 (4)	0.2646 (2)	0.29319 (11)	0.0292 (4)	
C4	0.3691 (4)	0.3697 (2)	0.38301 (12)	0.0315 (4)	
C5	0.3203 (4)	0.3700 (2)	0.18790 (12)	0.0337 (4)	
H5	0.4147	0.5070	0.1738	0.040*	
C6	0.2312 (4)	0.2720 (3)	0.10404 (12)	0.0355 (4)	
H6	0.2640	0.3426	0.0334	0.043*	
C7	0.0913 (4)	0.0659 (2)	0.12647 (12)	0.0315 (4)	
N1	−0.0103 (4)	−0.0518 (2)	0.04644 (9)	0.0372 (4)	
O1	0.2870 (4)	0.2717 (2)	0.47782 (9)	0.0545 (4)	
H1A	0.3524	0.3417	0.5217	0.082*	
O2	0.5255 (3)	0.54435 (18)	0.36449 (9)	0.0453 (4)	

### Atomic displacement parameters (Å^2^)

**Table:**

	*U*^11^	*U*^22^	*U*^33^	*U*^12^	*U*^13^	*U*^23^
C1	0.0452 (10)	0.0306 (9)	0.0329 (9)	−0.0099 (7)	−0.0026 (7)	−0.0095 (7)
C2	0.0438 (10)	0.0360 (9)	0.0243 (8)	−0.0081 (8)	−0.0014 (7)	−0.0072 (7)
C3	0.0321 (9)	0.0322 (8)	0.0261 (8)	−0.0037 (7)	−0.0030 (6)	−0.0115 (7)
C4	0.0377 (9)	0.0334 (9)	0.0253 (8)	−0.0060 (7)	−0.0040 (6)	−0.0092 (7)
C5	0.0436 (10)	0.0295 (8)	0.0299 (9)	−0.0082 (7)	−0.0009 (7)	−0.0092 (7)
C6	0.0465 (10)	0.0376 (9)	0.0240 (8)	−0.0067 (7)	−0.0030 (7)	−0.0084 (7)
C7	0.0333 (9)	0.0349 (9)	0.0299 (9)	−0.0023 (7)	−0.0048 (7)	−0.0144 (7)
N1	0.0471 (8)	0.0382 (8)	0.0300 (7)	−0.0081 (7)	−0.0058 (7)	−0.0143 (6)
O1	0.0875 (10)	0.0544 (8)	0.0254 (6)	−0.0314 (7)	−0.0023 (6)	−0.0128 (6)
O2	0.0674 (8)	0.0397 (7)	0.0321 (7)	−0.0215 (6)	−0.0027 (6)	−0.0119 (5)

### Geometric parameters (Å, °)

**Table:**

C1—C7	1.377 (2)	C4—O1	1.2800 (18)
C1—C2	1.389 (2)	C5—C6	1.381 (2)
C1—H1	0.9300	C5—H5	0.9300
C2—C3	1.384 (2)	C6—C7	1.396 (2)
C2—H2	0.9300	C6—H6	0.9300
C3—C5	1.388 (2)	C7—N1	1.4327 (19)
C3—C4	1.485 (2)	N1—N1^i^	1.239 (2)
C4—O2	1.246 (2)	O1—H1A	0.8200
			
C7—C1—C2	119.91 (16)	C6—C5—C3	120.22 (15)
C7—C1—H1	120.0	C6—C5—H5	119.9
C2—C1—H1	120.0	C3—C5—H5	119.9
C3—C2—C1	119.70 (15)	C5—C6—C7	119.22 (15)
C3—C2—H2	120.2	C5—C6—H6	120.4
C1—C2—H2	120.2	C7—C6—H6	120.4
C2—C3—C5	120.28 (14)	C1—C7—C6	120.67 (14)
C2—C3—C4	119.73 (14)	C1—C7—N1	115.05 (15)
C5—C3—C4	119.99 (15)	C6—C7—N1	124.28 (14)
O2—C4—O1	123.10 (14)	N1^i^—N1—C7	114.04 (17)
O2—C4—C3	120.27 (14)	C4—O1—H1A	109.5
O1—C4—C3	116.63 (15)		

Symmetry codes: (i) −*x*, −*y*, −*z*.

### Hydrogen-bond geometry (Å, °)

**Table:**

*D*—H···*A*	*D*—H	H···*A*	*D*···*A*	*D*—H···*A*
O1—H1A···O2^ii^	0.82	1.81	2.6181 (17)	170

Symmetry codes: (ii) −*x*+1, −*y*+1, −*z*+1.
